# Mortality in Thai Nursing Homes Based on Antimicrobial-Resistant *Enterobacterales* Carriage and COVID-19 Lockdown Timing: A Prospective Cohort Study

**DOI:** 10.3390/antibiotics11060762

**Published:** 2022-06-02

**Authors:** Thundon Ngamprasertchai, Muthita Vanaporn, Sant Muangnoicharoen, Wirichada Pan-ngum, Narisa Ruenroengbun, Pittaya Piroonamornpun, Thitiya Ponam, Chatnapa Duangdee, Phanita Chankete, Anupop Jitmuang, Visanu Thamlikitkul

**Affiliations:** 1 Department of Clinical Tropical Medicine, Faculty of Tropical Medicine, Mahidol University, Bangkok 10400, Thailand; sant.mua@mahidol.ac.th; 2 Department of Microbiology and Immunology, Faculty of Tropical Medicine, Mahidol University, Bangkok 10400, Thailand; muthita.van@mahidol.ac.th (M.V.); teerarut.cha@mahidol.ac.th (P.C.); 3 Department of Tropical Hygiene, Faculty of Tropical Medicine, Mahidol University, Bangkok 10400, Thailand; wirichada.pan@mahidol.ac.th; 4 Department of Pharmaceutics (Clinical Pharmacy), Faculty of Pharmacy, Slipakorn University, Nakornprathom 73000, Thailand; polladew@gmail.com; 5 Hospital for Tropical Diseases, Faculty of Tropical Medicine, Mahidol University, Bangkok 10400, Thailand; pittaya.pir@mahidol.ac.th (P.P.); thitiya.pon@mahidol.ac.th (T.P.); chatnapa.dua@mahidol.ac.th (C.D.); 6 Department of Medicine, Faculty of Medicine Siriraj Hospital, Mahidol University, Bangkok 10700, Thailand; anupopmix@yahoo.co.th (A.J.); visanu.tha@mahidol.ac.th (V.T.)

**Keywords:** *Enterobacterales*, colonization, nursing home, COVID-19, lockdown

## Abstract

Antimicrobial-resistant *Enterobacterales* carriage and the coronavirus disease 2019 (COVID-19) lockdown measures may impact the incidence all-cause mortality rate among nursing home residents. To determine the all-cause mortality rate in the presence/absence of antimicrobial-resistant *Enterobacterales* carriage and the incidence all-cause mortality rate before and during COVID-19 pandemic lockdown, this prospective closed-cohort study was conducted at various types of nursing homes in Bangkok, Thailand, from June 2020 to December 2021. The elderly residents included 142 participants (aged ≥60 years) living in nursing homes ≥3 months, who did not have terminal illnesses. Time-to-event analyses with Cox proportional hazards models and stratified log-rank tests were used. The all-cause mortality rate was 18%, and the incidence all-cause mortality rate was 0.59/1000 person-days in residents who had antimicrobial-resistant *Enterobacterales* carriage at baseline. Meanwhile, the incidence all-cause mortality rate among noncarriage was 0.17/1000 person-days. The mortality incidence rate of carriage was three times higher than residents who were noncarriage without statistical significance (HR 3.2; 95% CI 0.74, 13.83). Residents in nonprofit nursing homes had a higher mortality rate than those in for-profit nursing homes (OR 9.24; 95% CI 2.14, 39.86). The incidence mortality rate during and before lockdown were 0.62 and 0.30, respectively. Effective infection-control policies akin to hospital-based systems should be endorsed in all types of nursing homes. To limit the interruption of long-term chronic care, COVID-19 prevention should be individualized to nursing homes.

## 1. Introduction

Residents in nursing homes are favorable carriers of microbes [1] and spread antibiotic-resistant microbes, leading to antimicrobial resistance (AMR) [2]. Previous studies have shown that 4.7–64% of the residents of nursing homes had multidrug-resistant Gram-negative bacteria [3,4,5,6] that were subsequently transmitted to and infected [7] other patients or healthcare workers [8]. Although nursing homes provide limited healthcare services and have fewer beds than healthcare facilities, the probability of transmission of antimicrobial-resistant pathogens remains high [2]. Overuse and prolonged empirical administration of antimicrobial agents accompanied by poor infection-control policies increase AMR burden. [9] Furthermore, ineffective infection-control procedures resulted in the outbreak of the COVID-19 pandemic in nursing homes [10]. Therefore, nursing homes must improve their infection-control policies.

Thailand is experiencing a rapid increase in its aging population. One report estimated that the number of elderly persons aged ≥80 years would increase from 1.9 to 3 million persons in the from 2020 to 2040 [11]. Consequently, there has been an expansion of long-term care services (also known as nursing homes) in Thailand [12]. However, there are suboptimal standards of care offered, staff competencies, and ineffective systems within long-term care services [13]. Additionally, research regarding AMR bacteria situations in Thai nursing homes are limited. In community settings, 52.1–58.2% of Thai healthy volunteers had CTX-M beta-lactamase-producing *Enterobacterales* (EC) carriage [14,15]. We postulate that carriage rates in nursing homes have been described as a proxy composite indicator of AMR in the community [16]. The mortality rate of nursing home residents is determined by several factors, including advanced age, the presence of comorbidities, and having impaired cognitive or physical function or sarcopenia [17,18,19]. The outcomes resulting from AMR carriage or infection in nursing homes are diverse. Some studies showed lower mortality rates among residents without AMR carriage when compared with residents with AMR carriage; other studies showed similar mortality outcomes in the two groups [20,21]. However, Aliyu S et al. illustrated worse outcomes among residents infected with multidrug-resistant organisms [22]. We hypothesize that nursing home residents with AMR carriage are likely to develop an infection from their antimicrobial-resistant colonies for which they may receive inappropriate empirical antimicrobial agents. We also hypothesize that residents with AMR carriage would have a higher mortality rate than those with AMR noncarriage.

The COVID-19 pandemic not only affected nursing home residents but also impacted on the healthcare delivery system. In western countries, more than one-fourth of the documented deaths due to COVID-19 were reported among nursing home residents [23]. At the end of 2020, the number of COVID-19 cases in Thailand gradually increased but were successfully controlled. Nursing home policies were changed to limit the influx of residents and visiting family members. Henceforth, until April 2021, the number of community COVID-19 cases increased, resulting in the imposition of travel restrictions and social distancing measures in May 2021. Subsequently, all nursing homes were in full lockdown; thus, residents were unable to see their families or visit doctors in charge of their chronic illnesses. Although healthcare delivery was moved to telehealth platforms, the impact of the pandemic-related transition and patient outcomes has not yet been established.

We set out to compare the all-cause mortality rate among Thai nursing home residents with and without AMR-EC carriage. We also compared the all-cause mortality rate before and after the countrywide COVID-19 pandemic lockdown measures were imposed among Thai nursing home residents. Additionally, we described the risk factors for AMR-EC carriage and all-cause mortality among nursing home residents.

## 2. Results

Table 1 demonstrates the baseline characteristics of nursing home residents. We included 142 residents in total, with minimal loss to follow-up (5; 3.5%). Residents with multiple comorbidities or retaining catheter or having incontinence or pressure ulcer were frequently found AMR-EC carriage. The definitions of study variables were available in material and method part.

### 2.1. Characteristics and Factors Associated EC Carriage

Nearly 74% of residents had AMR-EC carriage at enrollment (Table 1). Almost 38% of residents had 3GCR-EC carriage, and 70% had QREC carriage. *E. coli* was the most isolated EC (68.6%) (Supplementary Materials Table S1). No carbapenem-resistant EC was found in our study population. Factors associated with AMR-EC carriage at enrollment and QREC carriage were incontinence or pressure ulcer (OR 3.44; 95% CI 1.43, 8.30), foreign materials or retained catheters (OR 3.62; 95% CI 1.45, 9.05), and multiple comorbidities (OR 17.33; 95% CI 1.63, 184.36). Elderly residents who had been living in nursing homes for more than 1 year developed 3GCR-EC less than those who had been living in nursing homes for less than 1 year (OR 0.46; 95% CI 0.22, 0.96). After confounding adjustment, elderly residents who had incontinence or existing pressure ulcers (OR 2.15; 95% CI 0.75, 6.18) and a catheter or retained foreign material (OR 2.30; 95% CI 0.77, 6.90) were more likely to have AMR-EC carriage (Table 2).

### 2.2. Mortality Rate of EC Carriage

**Figure 1:**
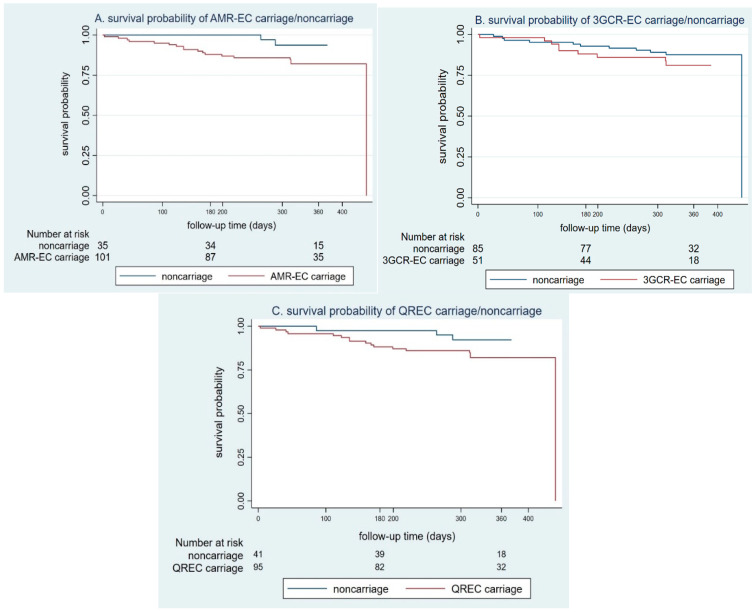
The survival probability between carriage (red line) and noncarriage (blue line) antimicrobial-resistant *Enterobacterales* stratified by types of resistance: (**A**) AMR-EC (*p* value log-rank 0.10); (**B**) 3GCR-EC (*p* value log-rank 0.32); (**C**) QREC (*p* value log-rank 0.14).

The all-cause mortality rate in residents who had AMR-EC carriage at baseline was approximately 18%; similar rates were seen in residents with 3GCR-EC and QREC carriage (Supplementary Materials Table S2). The incidence all-cause mortality rate among residents with AMR-EC carriage was 0.59 per 1000 person-days (95% CI 0.37, 0.93). The mortality incidence rate among residents with AMR-EC carriage was thrice that of those with AMR-EC noncarriage without statistical significance (HR 3.2; 95% CI 0.74, 13.83). The Kaplan–Meier curve (Figure 1) represented survival probability between residents who were carriage at enrollment and those who were noncarriage showed that noncarriage had higher survival probability than carriage by all types of resistance without statistical significance. The hazard ratios of AMR carriage have a similar trend in residents with 3GCR-EC (HR 1.57; 95% CI 0.64, 3.87) and QREC (HR 2.45, 95% CI 0.71, 8.42) without statistical significance.

### 2.3. Associated Factors and Incidence Mortality Rate among Residents

**Figure 2:**
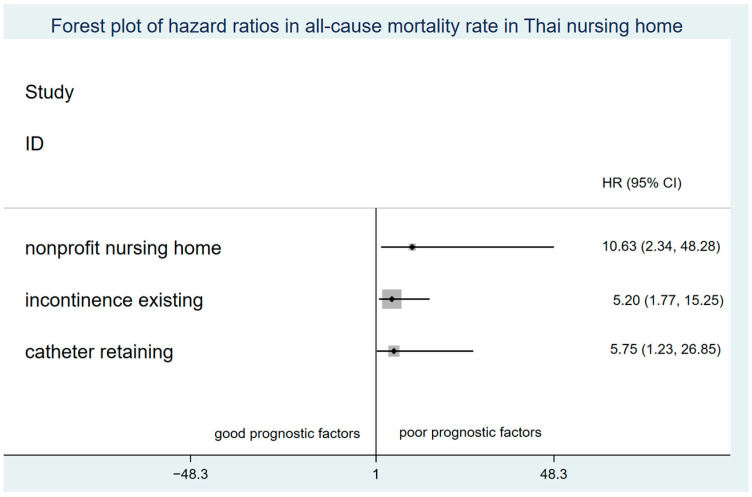
Forest plot of adjusted hazard ratios in all-cause mortality rate in Thai nursing homes.

The overall mortality rate among residents who had been living in nursing homes at the 1-year follow-up date was 14.8%. The mortality rate before and during the lockdown was approximately 4% and 11%, respectively (Supplementary Materials Table S3). The incidence mortality rate during the lockdown period (0.62; 95% CI 0.37, 1.02) was double that before the lockdown period (0.30; 95% CI 0.14, 0.67). The Kaplan–Meier curve (Supplementary Materials Figure S1) shows mortality probability before and during the COVID-19 pandemic lockdown. Only one case was mortality reported because of COVID-19 infection during the lockdown period. After confounding adjustments, the factors of nonprofit nursing home, incontinence, pressure ulcer existing, and catheter or foreign material retaining were poor prognostic factors for nursing home residents (Figure 2). Supplementary Materials Figure S2 showed that residents in nonprofit homes had the lowest survival probability when compared with residents in for-profit nursing homes (*p* < 0.001). Dependent residents had the worst survival outcome when compared with more able residents (Supplementary Materials Figure S3) (*p* = 0.004) and had a mortality rate that was triple that of partially dependent residents (OR 3.71; 95% CI 1.43, 9.67). We performed a global test to check the constant of the coefficient over time; *p* value was 0.326, which was represented the validity of the assumption.

## 3. Discussion

In this cohort of 142 nursing home residents, the all-cause mortality rate in 1 year follow-up was 14.8%, which was relatively lower than that in the literature, which was approximately 28–30% [17,19]. Only one death was caused by COVID-19. Residents with AMR-EC, 3GCR-EC, and QREC had a mortality rate of approximately 17.8%, which was also lower than in previous studies which approximately was 25–50% [8,20,24]; meanwhile, the mortality rate among noncarriage was approximately 5.7–13%. Our results concurred with the results in the literature regarding the incidence all-cause mortality rate among persons with drug-resistant carriage that is usually higher than in persons with noncarriage without statistical significance, i.e., almost triple, as they developed infection from their antimicrobial-resistant colonies. These may result from infection after the carriage and inappropriate empirical antimicrobial use; however, previous reports have demonstrated controversial results regarding the clinical significance of carriage [8,20,24,25]. Effective infection-control strategies such as hand hygiene or the use of standard personal protective equipment similar to that practiced in hospitals should be enforced in nursing homes. Residents who are likely to be carriers, for example, of poor ADLs status or with the presence of risk factors for AMR-EC, should be isolated in a cohort ward. Only trained staff should perform standard nursing care. We found that residents who had been living in a nursing home for more than 1 year were less likely to develop 3GCR-EC carriage. This implies that carriage would be substituted by noncarriage over time if there was no antimicrobial pressure effect [26]. We therefore recommend antimicrobial stewardship programs in nursing homes to reduce AMR-EC carriage. The 3GCR-EC carriage rate was less than that found in the community [14,15], since most of the residents have lived in nursing homes for more than 1 year. Overall, AMR-EC carriage in Thai nursing homes was higher than in the previous studies [8,27,28], which were either conducted in high-income countries or had different AMR pathogens other than EC.

The COVID-19 pandemic has had a devastating effect on nursing home residents in many countries, but our study illustrated favorable results. Although there was no COVID-19 outbreak in our cohort, the incidence all-cause mortality rate during the COVID-19 pandemic was double that before the lockdown. All their family members and their doctors’ meetings were transposed to a virtual platform. Nearly 70% of our cohort residents who needed regular follow-up had their care interrupted during the COVID-19 pandemic lockdowns. With telehealth, essential physical examinations cannot be done by a doctor, which may lead to an incorrect or a missed diagnosis. It is possible that the aggravation of their underlying chronic illnesses or missed diagnoses were the main causes of death during the lockdown period. Though telehealth will not solve this situation all, it is appropriate in settings which infrastructure remains intact and doctors are able to see patients [29]. Flexible and appropriate COVID-19 prevention policies should be individualized in diverse settings. There was an unequal mortality rate in different nursing homes; an attribute related to nursing home quality ratings. Highly rated nursing homes had lower death rates, as well as fewer cases of COVID-19 [30,31,32]. Nonprofit nursing homes had the worst survival probability among nursing homes since they did not offer doctor-provided care, which the hospital-based and private nursing homes did. Although residents in hospital-based nursing homes tend to develop drug-resistant carriage and are more likely to have severe chronic diseases, hospital settings implement standardized nursing care and appropriate antimicrobial use. We recommend that the Ministry of Public Health endorse standardized nursing care akin to that in hospital-based nursing homes to improve the quality of life and the survival outcomes of residents in nonprofit nursing homes. Furthermore, the COVID-19 pandemic has also affected the health service, controlling AMR at both the hospital level and the community level [33]. Increasing antimicrobial usage and infection-control system interruption might impact on AMR situation in near future.

Our study had several strengths. First, we included residents from different types of nursing homes to enhance the generalizability of our results. Additionally, we prospectively followed up our participants and had a minimal loss to follow-up. Lastly, we were able to establish the impact of moving to telehealth, strict infection-control policies, and the inequality experienced by residents of different types of nursing homes. However, some study limitations must be addressed. First, our results regarding the association between AMR-EC carriage and mortality did not attain statistical significance. This could be due to our limited sample size. Second, we assessed for AMR-EC carriage only at one point during enrollment; our results may have been more robust if we had prospectively measured AMR-EC carriage over the whole study period. Longitudinal AMR-EC surveillance study may be needed to understand natural progression of carriage/noncarriage. Lastly, we performed COVID-19 testing in participants who were symptomatic when they first arrived at a nursing home; thus, asymptomatic residents who may have had COVID-19 were not be captured by our study.

## 4. Materials and Methods

### 4.1. Study Design and Setting

This prospective closed-cohort study was conducted from June 2020 to December 2021. The enrolment date began on 1st June 2020 and followed up till 30th April 2021, which was the beginning of COVID-19 lockdown. Then, we followed up with participants 6 months afterward. Our cohort nursing home limited new members of residents before COVID-19 lockdown. However, during lockdown, all residents who went outside nursing homes for any reason were tested and isolated. All their family members and their doctors’ meetings were transposed to a virtual platform. We recruited residents living in six different types of nursing homes in Bangkok. We collected data from three for-profit nursing homes: one hospital-based nursing home located in and run by the Hospital for Tropical Diseases, Faculty of Tropical Medicine, Mahidol University; and two private centers run by the private sector. We also collected data from three nonprofit nursing homes.

### 4.2. Study Populations

We enrolled residents, aged ≥60 years without a terminal illness or illnesses, who had been living in nursing homes for at least 3 months preceding the interview date. Exclusion criteria included residents who developed end-of-life conditions or there was tendency to be lost to follow-up within 1 year after enrollment. Participants for whom rectal sampling also was contraindicated were excluded. 

### 4.3. Study Procedure and Data Collection 

#### 4.3.1. Rectal Sampling and Microbiological Outcome Measurement

We assessed EC carriage using rectal swabs since they are easier to collect than stool samples, and results obtained from rectal swabs are highly correlated with those obtained from stool samples [34]. Rectal sampling was at enrollment performed by trained investigators. Swabs were placed on BBL^TM^ CultureSwab^TM^ EZ II (COPAN ITALIA SpA, Brescia, Italy) at 25 °C. Samples were then sent to the microbiology laboratory for identification and drug-susceptible testing at the Hospital for Tropical Diseases and Department of Microbiology and Immunology at the Faculty of Tropical Medicine, Mahidol University, the same day they were collected. The target bacteria, EC, were identified using different media and standard biochemical conventional methods. Antimicrobial susceptibility testing (AST) was performed via disk diffusion method and interpreted according to the standard recommendation of the Clinical Laboratory Standard Institute 2021-2.

#### 4.3.2. Study Endpoints

The primary endpoint was the incidence all-cause mortality rate in nursing home residents with or without AMR-EC. The secondary endpoint was the incidence all-cause mortality rate in nursing home residents before and during the COVID-19 pandemic lockdown. All-cause mortality was defined as death due to any cause during the entire duration of the study. Factors associated with all-cause mortality and AMR-EC carriage were also described. Prevalence, type, and pattern of carriage among residents were also defined. This study was registered in the Thai Clinical Trials Registry on 2 June 2019, with ID number (TCTR20190602003). In this study, we follow the Strengthening the Reporting of Observational Studies in Epidemiology reporting guidelines.

#### 4.3.3. Study Variables Definition

Definition of targeted isolated bacteria were EC-categorized based on the type of drug resistance as follows: quinolone resistant EC (QREC) defined as EC resistant to either ciprofloxacin or levofloxacin or both, third-generation cephalosporin-resistant EC (3GCR-EC) were resistant to either ceftazidime or cefotaxime or both, and AMR-EC defined as EC that were either QREC or 3GC-EC or both. Carriage/noncarriage was defined as the presence/absence of AMR-EC was in rectal swabs, respectively.

We selected factors that were clinically meaningful to determine mortality rate and AMR-EC carriage. List of factors [8,17,18,19,27,35] were as follows; The age group was classified to be less than 80 years, or equal to and more than 80 years, based on the mean age of our study population; this age was also referred to when determining the mortality risk [36]. Comorbidities were categorized into single or multiple comorbidities; the duration of living in a nursing home was classified into two: <1 or ≥1 year. Physical function was measured using activities of daily living (ADLs) measures, which were grouped into dependence, partial independence, and independence. We defined dependence as being unable to perform basic ADLs, an ability to do instrumental ADLs without any assistance as independence, and the need for a caregiver to assist in instrumental ADLs as partial dependence. Incontinence was categorized as urinary or fecal incontinence. Information about the presence or absence of pressure ulcers was also collected. The use of catheters or internally retained foreign materials, such as urinary catheters, nasogastric tubes, venous or arterial catheters, and pacemakers, and having a tracheostomy were considered risk factors for AMR-EC carriage and mortality in our analyses. Some residents had to regularly visit a hospital for follow-up of a diagnosed chronic health condition and to receive their long-term medications. 

### 4.4. Statistical Method

#### 4.4.1. Sample Size Calculation

From previous literature, the mortality rate in noncarriage and carriage AMR-EC nursing home residents was 12–30% and 25–50%, respectively [8,20,24]. We estimated that the mortality rate among noncarriage and carriage AMR-EC would be 15% and 35%, respectively. The prevalence of the CTX-M beta-lactamase-producing EC in healthy volunteers in Thailand is 50% [14,15]; therefore, the ratio of the population with carriage and noncarriage is 1:1. From this, we aimed to recruit 73 participants in each group with a power of 80% and an α error of 0.05%.

#### 4.4.2. Statistical Analysis

To identify risk factors for AMR-EC carriage, odds ratios (OR) and 95% confidence interval (CI) were computed using logistic regression analysis. All predictors with a *p* value of <0.10 in univariate analysis were included in multiple logistic regression models with stepwise forward selection. We used the time-to-events analyses (Kaplan–Meier) and stratified log-rank tests to compare time to death between the carriage and noncarriage groups. We used Cox proportional hazards models to describe factors associated with mortality, which were reported as HRs with 95% confidence intervals (CIs). The date of censoring was 30 April 2021 as COVID-19 lockdown and 6 months afterward. The incidence mortality rate was computed as the number of deaths per population per 1000 days living in a nursing home. The person-days used for AMR-EC mortality were calculated using the duration between the date of enrollment and the date of follow-up. Person-days before the COVID-19 pandemic lockdown were taken as the duration between the date of enrollment and the date of lockdown, 1 April 2021; conversely, person-days after the lockdown were taken as the duration between the date of lockdown and date of follow-up. The global test was used to check the constant of the coefficient over time. The data were analyzed using StataBE v17.0 software (StataCorp, College Station, TX, USA); *p* values of <0.05 were considered two-sided and statistically significant.

## 5. Conclusions

The incidence all-cause mortality rate among nursing home residents with AMR-EC carriage was higher than that in residents with noncarriage without statistical significance. Standard nursing care and effective infection-control policies similar to those in hospital-based systems should be endorsed in all types of nursing homes. Nursing home residents with chronic diseases whose mandatory long-term follow-up was interrupted during the COVID-19 pandemic had a higher mortality rate. The COVID-19 pandemic also affected health service’s control of AMR at both the hospital level and the community level. Increasing antimicrobial usage and infection-control system interruption might impact on AMR situation in near future. Eventually, COVID-19 prevention policies should be individualized based on the type of nursing home.

## Figures and Tables

**Table 1:** Baseline and characteristics of antimicrobial-resistant *Enterobacterales* carriage of Thai nursing home residents at study enrolment.

Characteristics	Residents *N* = 142, *N*. (%)	*Enterobacterales* Carriage * (*N* = 136)		
		Antimicrobial-Resistant Carriage (*N* = 101; 74.3%), No. (%)	No Antimicrobial-Resistant Carriage (*N* = 35; 25.8%) No. (%)	*p* Value
**Sex** **Male** **Female**	57 (40.1) 85 (59.9)	37 (36.6) 64 (63.4)	19 (54.3) 16 (45.7)	0.067
**Age, year** <80 ≥80	67 (47.2) 75 (52.8)	49 (48.5) 52 (51.5)	14 (40.0) 21 (60.0)	0.384
**Comorbidities** None Single Multiple	5 (3.5) 33 (23.2) 104 (73.2)	1 (1) 26 (25.7) 74 (73.3)	4 (11.4) 6 (17.1) 25 (71.4)	0.014 ^+^
**Duration of living, year** <1 ≥1	46 (32.4) 96 (67.6)	33 (32.7) 68 (67.3)	11 (31.4) 24 (68.6)	0.892
**Types of nursing home** Nonprofit Profit	73 (51.4) 69 (48.6)	56 (55.5) 45 (44.6)	15 (42.9) 20 (57.1)	0.199
**Activities of Daily Living** **(ADLs)** Dependence Partial dependence Independence	53 (37.6) 76 (53.9) 12 (8.5)	39 (39.0) 56 (56.0) 5 (5.0)	12 (34.3) 17 (48.6) 6 (17.1)	0.078
**Incontinence or pressure** **ulcer existing** Yes No	62 (43.7) 80 (56.3)	51 (50.5) 50 (49.5)	8 (22.9) 27 (77.1)	0.016 ^+^
**Catheter or foreign** **material retaining** Yes No	59 (41.6) 83 (58.5)	48 (47.6) 53 (52.5)	7 (20.0) 28 (80.0)	0.016^+^
**Recent antimicrobial** **agents use** Yes No	50 (38.2) 81 (61.8)	39 (41.1) 56 (59.0)	8 (26.7) 22 (73.3)	0.156
**Need regular** **follow-up** Yes No	60 (72.2) 23 (27.7)	41 (68.3) 18 (30.0)	14 (70.0) 4 (20.0)	0.189
**Recent in hospital** **admission** Yes No	41 (49.3) 42 (50.6)	28 (46.7) 31 (51.7)	9 (45.0) 11 (55.0)	0.829
**Recent ICU admission** Yes No	8 (22.2) 28 (77.8)	6 (25.0) 18 (75.0)	1 (11.1) 8 (88.9)	0.385

+ statistical significance; * Carriage/noncarriage was defined as the presence/absence of AMR-EC was in rectal swabs, respectively.

**Table 2:** Factors associated with antimicrobial-resistant *Enterobacterales* carriage of Thai nursing home residents at study enrolment.

Characteristics	Quinolone-Resistant EC * Carriage		Third-Generation Cephalosporin-Resistant EC ** Carriage		Antimicrobial Resistant EC *** Carriage	
	Crude OR (95% CI)	Adjusted OR (95%CI)	Crude OR (95% CI)	Adjusted (OR 95% CI)	Crude OR (95% CI)	Adjusted OR (95%CI)
**Comorbidities** None Single Multiple	- 1 12 (1.16,123.68)				- 1 17.33 (1.63,184.36)	
**Duration of living, year** <1 ≥1			1 0.46 (0.22,0.96)			
**Activities of Daily Living (ADLs)** Dependence Partial dependence Independence	4.63 (1.17,18.27) 4.64 (1.22, 17.57) 1	0.96 (0.19,4.91) 3.52 (0.92,13.45) 1				
**Incontinence or pressure ulcer existing** Yes No	3.95 (1.70, 9.17) 1				3.44 (1.43, 8.30) 1	2.15 (0.75, 6.18) 1
**Catheter or foreign material retaining** Yes No	4.96 (2.00, 12.29) 1	9.52 (2.74,33.11) 1			3.62 (1.45, 9.05) 1	2.30 (0.77, 6.90) 1
**Recent antimicrobial agents use** Yes No	2.437 (1.00, 5.96) 1					

* Quinolone resistant *Enterobacterales* (EC) defined as EC resistant to either ciprofloxacin or levofloxacin or both; ** third-generation cephalosporin-resistant EC (3GCR-EC) defined as EC were resistant to either ceftazidime or cefotaxime or both; *** antimicrobial resistant (AMR) EC defined as EC were either QREC or 3GC-EC or both.

## Data Availability

Not applicable.

## Supplementary Materials

The following supporting information can be downloaded at: https://www.mdpi.com/article/10.3390/antibiotics11060762/s1, Table S1: Microbiology results at enrollment (isolations), Table S2: Incidence all-cause mortality rate among antimicrobial-resistant *Enterobacterales* carriage, Table S3: Comparison of incidence all-cause mortality rate by COVID-19 pandemic lockdown, Figure S1: The mortality probability before COVID-19 lockdown (A) and during COVID-19 lockdown (B), Figure S2: The survival probability between types of nursing home (blueline: hospital based (profit) nursing home; red line: private (profit) nursing home; green line: non-profit nursing home) (*p* value log-rank <0.001), Figure S3: The survival probability between types of Activities Daily Living (ADLs) (blue line: dependence; red line: partial dependence; green line: independence) (*p* value log-rank 0.004).

## References

[1] Aschbacher R., Pagani E., Confalonieri M., Farina C., Fazii P., Luzzaro F., Montanera P.G., Piazza A., Pagani L. (2016). Review on colonization of residents and staff in Italian long-term care facilities by multidrug-resistant bacteria compared with other European countries. Antimicrob. Resist. Infect. Control.

[2] van den Dool C., Haenen A., Leenstra T., Wallinga J. (2016). The Role of Nursing Homes in the Spread of Antimicrobial Resistance over the Healthcare Network. Infect. Control Hosp. Epidemiol..

[3] Ruscher C., Pfeifer Y., Layer F., Schaumann R., Levin K., Mielke M. (2014). Inguinal skin colonization with multidrug-resistant bacteria among residents of elderly care facilities: Frequency, persistence, molecular analysis and clinical impact. Int. J. Med Microbiol..

[4] March A., Aschbacher R., Dhanji H., Livermore D.M., Böttcher A., Sleghel F., Maggi S., Noale M., Larcher C., Woodford N. (2010). Colonization of residents and staff of a long-term-care facility and adjacent acute-care hospital geriatric unit by multiresistant bacteria. Clin. Microbiol. Infect..

[5] Rooney P.J., O’Leary M.C., Loughrey A.C., McCalmont M., Smyth B., Donaghy P., Badri M., Woodford N., Karisik E., Livermore D.M. (2009). Nursing homes as a reservoir of extended-spectrumβ-lactamase (ESBL)-producing ciprofloxacin-resistant Escherichia coli. J. Antimicrob. Chemother..

[6] Cochard H., Aubier B., Quentin R., van der Mee-Marquet N., du Centre R.D.H. (2014). Extended-Spectrumβ-Lactamase–Producing Enterobacteriaceae in French Nursing Homes: An Association between High Carriage Rate among Residents, Environmental Contamination, Poor Conformity with Good Hygiene Practice, and Putative Resident-to-Resident Transmission. Infect. Control Hosp. Epidemiol..

[7] Reddy P., Malczynski M., Obias A., Reiner S., Jin N., Huang J., Noskin G.A., Zembower T. (2007). Screening for Extended-Spectrum -Lactamase-Producing Enterobacteriaceae among High-Risk Patients and Rates of Subsequent Bacteremia. Clin. Infect. Dis..

[8] Leitner E., Zechner E., Ullrich E., Zarfel G., Luxner J., Pux C., Pichler G., Schippinger W., Krause R., Zollner-Schwetz I. (2018). Low prevalence of colonization with multidrug-resistant gram-negative bacteria in long-term care facilities in Graz, Austria. Am. J. Infect. Control.

[9] Nicolle L.E. (2000). Infection Control in Long-Term Care Facilities. Clin. Infect. Dis..

[10] Baker N.R., Dunn D., Greenberg S.A., Shaughnessy M. (2021). Infection Control in Long-Term Care: An Old Problem and New Priority. J. Am. Med. Dir. Assoc..

[11] Peae P. (2020). Situation of the Thai Elderly 2019.

[12] Lloyd-Sherlock P.G., Sasat S., Sanee A., Miyoshi Y., Lee S. (2021). The rapid expansion of residential long-term care services in Bangkok: A challenge for regulation. J. Public Health Dev..

[13] Sasat S., Choowattanapakorn T., Pukdeeprom T., Lertrat P., Aroonsang P. (2017). Long-Term Care Institutions in Thailand. J. Health Res..

[14] Niumsup P.R., Tansawai U., Na-Udom A., Jantapalaboon D., Assawatheptawee K., Kiddee A., Romgaew T., Lamlertthon S., Walsh T.R. (2017). Prevalence and risk factors for intestinal carriage of CTX-M-type ESBLs in Enterobacteriaceae from a Thai community. Eur. J. Clin. Microbiol..

[15] Sasaki T., Hirai I., Niki M., Nakamura T., Komalamisra C., Maipanich W., Kusolsuk T., Sa-Nguankiat S., Pubampen S., Yamamoto Y. (2010). High prevalence of CTX-M -lactamase-producing Enterobacteriaceae in stool specimens obtained from healthy individuals in Thailand. J. Antimicrob. Chemother..

[16] Thamlikitkul V., Tangkoskul T., Seenama C. (2019). Fecal Carriage Rate of Extended-Spectrum Beta-Lactamase-Producing Enterobacteriaceae as a Proxy Composite Indicator of Antimicrobial Resistance in a Community in Thailand. Open Forum Infect. Dis..

[17] Hjaltadóttir I., Hallberg I.R., Ekwall A.K., Nyberg P. (2011). Predicting mortality of residents at admission to nursing home: A longitudinal cohort study. BMC Health Serv. Res..

[18] Saka B., Ozkaya H., Karisik E., Akin S., Akpinar T., Tufan F., Bahat G., Dogan H., Horasan Z., Cesur K. (2016). Malnutrition and sarcopenia are associated with increased mortality rate in nursing home residents: A prospective study. Eur. Geriatr. Med..

[19] Vossius C., Selbæk G., Benth J., Bergh S. (2018). Mortality in nursing home residents: A longitudinal study over three years. PLoS ONE.

[20] Schoevaerdts D., Agelas J.-P., Ingels M.-G., Jamart J., Frennet M., Huang T.-D., Swine C., Glupczynski Y. (2013). Health outcomes of older patients colonized by multi-drug resistant bacteria (MDRB): A one-year follow-up study. Arch. Gerontol. Geriatr..

[21] Igbinosa O., Dogho P., Osadiaye N. (2019). Carbapenem-resistant Enterobacteriaceae: A retrospective review of treatment and outcomes in a long-term acute care hospital. Am. J. Infect. Control.

[22] Aliyu S., McGowan K., Hussain D., Kanawati L., Ruiz M., Yohannes S. (2021). Prevalence and Outcomes of Multi-Drug Resistant Blood Stream Infections among Nursing Home Residents Admitted to an Acute Care Hospital. J. Intensiv. Care Med..

[23] Grabowski D.C., Mor V. (2020). Nursing Home Care in Crisis in the Wake of COVID-19. JAMA.

[24] Choi J.-P., Cho E.H., Lee S.J., Koo M.S., Song Y.G. (2012). Influx of multidrug resistant, Gram-negative bacteria (MDRGNB) in a public hospital among elderly patients from long-term care facilities: A single-center pilot study. Arch. Gerontol. Geriatr..

[25] Ramphal R., Ambrose P.G. (2006). Extended-Spectrum β-Lactamases and Clinical Outcomes: Current Data. Clin. Infect. Dis..

[26] Östholmbalkhed Å., Tärnberg M., Nilsson M., Nilsson L.E., Hanberger H., Hällgren A., for the Southeast Sweden Travel Study Group (2018). Duration of travel-associated faecal colonisation with ESBL-producing Enterobacteriaceae—A one year follow-up study. PLoS ONE.

[27] Latour K., Huang T.-D., Jans B., Berhin C., Bogaerts P., Noel A., Nonhoff C., Dodémont M., Denis O., Ieven M. (2019). Prevalence of multidrug-resistant organisms in nursing homes in Belgium in 2015. PLoS ONE.

[28] Lee C.-M., Lai C.-C., Chiang H.-T., Lu M.-C., Wang L.-F., Tsai T.-L., Kang M.-Y., Jan Y.-N., Lo Y.-T., Ko W.-C. (2017). Presence of multidrug-resistant organisms in the residents and environments of long-term care facilities in Taiwan. J. Microbiol. Immunol. Infect..

[29] Hollander J.E., Carr B.G. (2020). Virtually Perfect? Telemedicine for COVID-19. N. Engl. J. Med..

[30] Williams C.S., Zheng Q., White A.J., Bengtsson A.I., Shulman E.T., Herzer K.R., Fleisher L.A. (2021). The association of nursing home quality ratings and spread of COVID-19. J. Am. Geriatr. Soc..

[31] Li Y., Temkin-Greener H., Shan G., Cai X. (2020). COVID-19 Infections and Deaths among Connecticut Nursing Home Residents: Facility Correlates. J. Am. Geriatr. Soc..

[32] He M., Li Y., Fang F. (2020). Is There a Link between Nursing Home Reported Quality and COVID-19 Cases? Evidence from California Skilled Nursing Facilities. J. Am. Med. Dir. Assoc..

[33] Rawson T.M., Ming D., Ahmad R., Moore L.S.P., Holmes A.H. (2020). Antimicrobial use, drug-resistant infections and COVID-19. Nat. Rev. Microbiol..

[34] Lerner A., Romano J., Chmelnitsky I., Navon-Venezia S., Edgar R., Carmeli Y. (2013). Rectal Swabs Are Suitable for Quantifying the Carriage Load of KPC-Producing Carbapenem-Resistant Enterobacteriaceae. Antimicrob. Agents Chemother..

[35] Suñer C., Ouchi D., Mas M., Alarcon R.L., Mesquida M.M., Prat N., Bonet-Simó J.M., Izquierdo M.E., Sánchez I.G., Noguerola S.R. (2021). A retrospective cohort study of risk factors for mortality among nursing homes exposed to COVID-19 in Spain. Nat. Aging.

[36] Panagiotou O.A., Kosar C.M., White E.M., Bantis L.E., Yang X., Santostefano C.M., Feifer R.A., Blackman C., Rudolph J.L., Gravenstein S. (2021). Risk Factors Associated with All-Cause 30-Day Mortality in Nursing Home Residents with COVID-19. JAMA Intern. Med..

