# Supplementary material for: Mortality in Thai Nursing Homes Based on Antimicrobial-Resistant Enterobacterales Carriage and COVID-19 Lockdown Timing: A Prospective Cohort Study
Source: Antibiotics (Basel). 2022 Jun 2;11(6):762. doi: 10.3390/antibiotics11060762 (PMC9219865; doi:10.3390/antibiotics11060762)
Supplement: Supplementary file 1 [file antibiotics-11-00762-s001.zip › antibiotics-1738826-supplementary.pdf]

Supplement materials

Table S1. Microbiology results at enrollment (isolations)

Enterobacterales	All isolation* (N=226) No. (%)	Quinolone resistance (N=136) No. (%)	3rd generation cephalosporin resistance (N=59) No. (%)
E. coli	155 (68.58)	102 (65.81)	48 (30.97)
Klebsiella spp.	31 (13.72)	13 (41.94)	4 (12.90)
Enterobacter spp.	13 (5.75)	7 (53.85)	2 (15.38)
Proteus spp.	21 (9.29)	12 (57.14)	4 (19.05)
Citrobacter spp.	6 (2.65)	2 (33.34)	1 (16.67)
**Overall**	226	136 (60.18)	59 (26.11)

*All isolations were tested for carbapenems resistance by susceptibility of ertapenem, imipenem, meropenem, and doripenem

Table S2. Incidence all-cause mortality rate among antimicrobial-resistant *Enterobacterales* carriage

Carriage	No. (N=136) (%)	Death (%)*	No of person-day (Per 1000 days)	HR (95% CI)	Incidence all-cause mortality rate (95% CI)
AMR-EC*	101 (74.3)	18 (17.8)	30,704	3.20 (0.74, 13.83)	0.59 (0.37, 0.93)
Non-AMR-EC	35 (25.7)	2 (5.7)	11,455	1 (reference)	0.17 (0.04, 0.70)
3GCR-EC **	51 (37.5)	9 (17.7)	15,415	1.57 (0.64, 3.87)	0.58 (0.30, 1.12)
Non 3GCR-EC	85 (62.5)	11 (13.0)	26,744	1 (reference)	0.41 (0.23, 0.74)
QREC***	95 (69.9)	17 (17.9)	28,882	2.45 (0.71, 8.42)	0.59 (0.37, 0.95)
Non QREC	41 (30.2)	3 (7.3)	13,277	1 (reference)	0.23 (0.07, 0.70)

*% per total carriage

Table S3. Comparison of incidence all-cause mortality rate by COVID-19 pandemic lockdown

Timing	No.	No. death (%)	No of person-day (per 1000 days)	Incidence all-cause mortality rate (95% CI)
**Pre COVID-19 pandemic lockdown**	142	6 (4.23)	19900	0.30 (0.14, 0.67)
**COVID-19 pandemic lockdown**	136	15 (11.03)	24275	0.62 (0.37, 1.02)
**Overall**	142	21 (14.79)	44175	0.48 (0.31, 0.73)

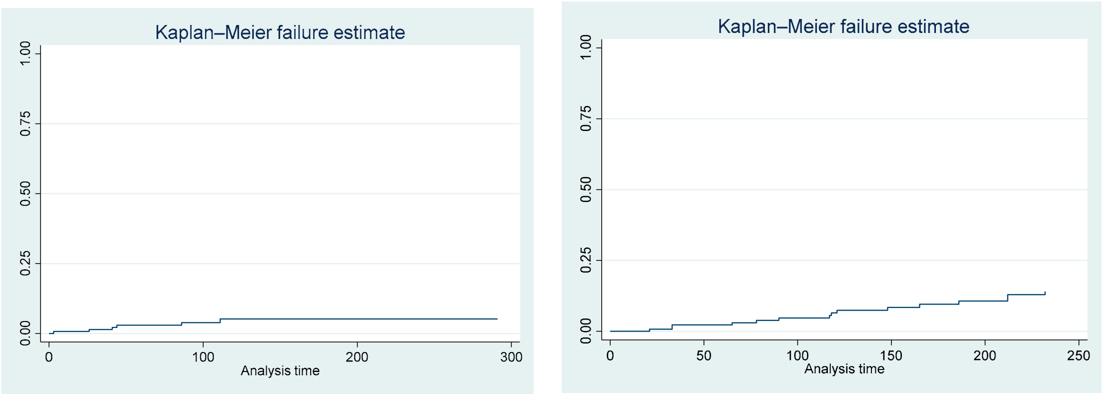

A. Before COVID-19 lockdown B. During COVID-19 lockdown

Figure S1. The mortality probability before COVID-19 lockdown (A) and during COVID-19 lockdown (B)

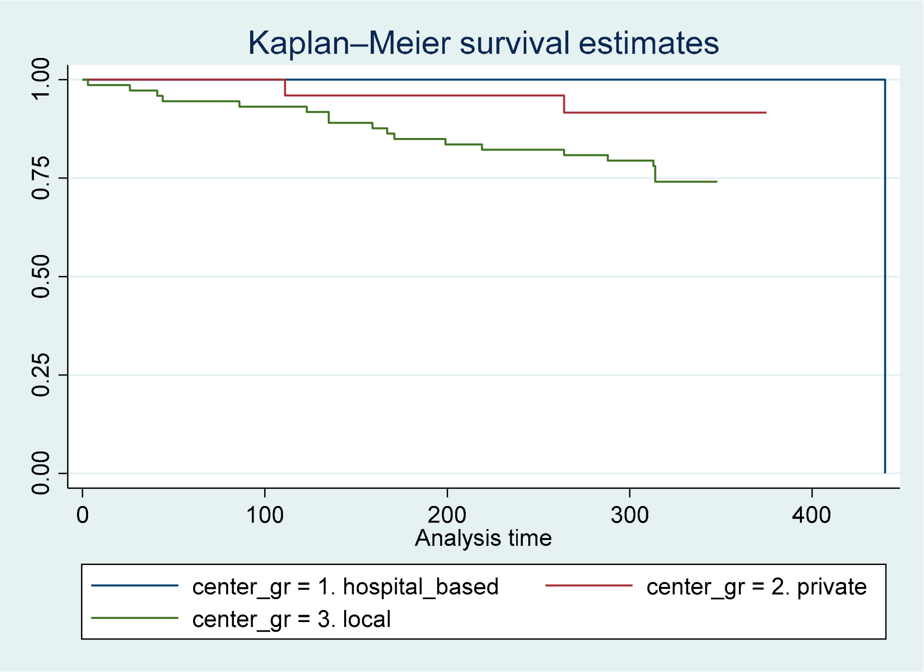

Figure S2. The survival probability between types of nursing home; Blueline: hospital based (profit) nursing home, red line: private (profit) nursing home, and green line: non-profit nursing home. (*p* value log rank <0.001)

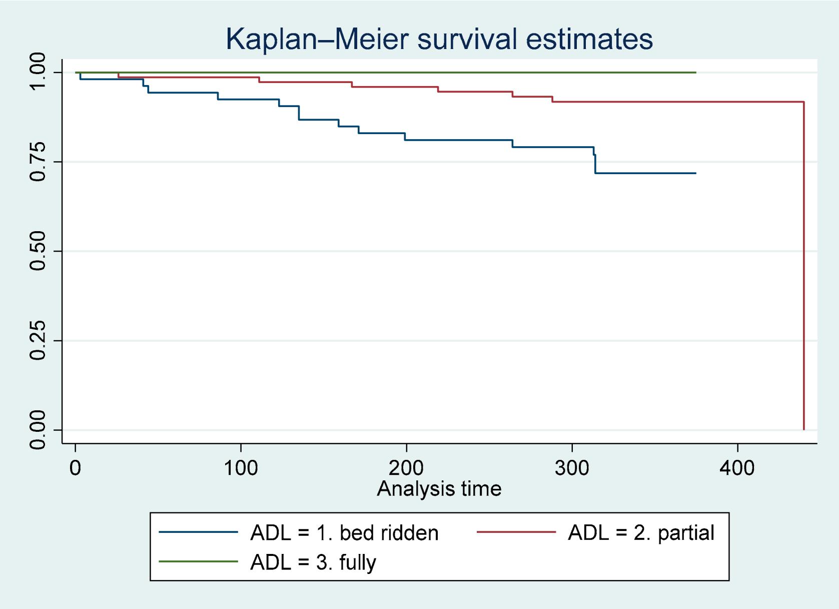

Figure S3. The survival probability between types of Activities Daily Living (ADLs); Blue line: dependence, red line: partial dependence, and green line: independence (*p* value log rank 0.004)
